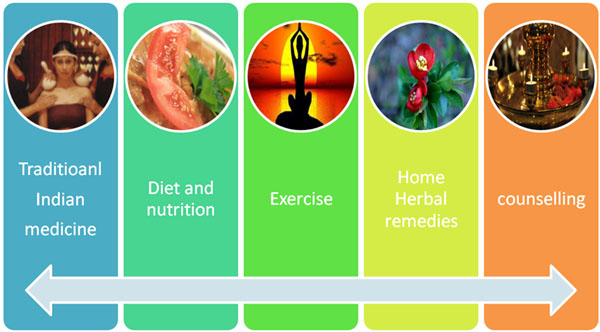# Effects of health education programme on teenagers with premenstrual syndrome

**DOI:** 10.1186/1878-5085-5-S1-A158

**Published:** 2014-02-11

**Authors:** Amina Ather

**Affiliations:** 1 Medizin Park Ruhr, Castrop-Rauxel, Germany

## Introduction

An health education programme was evaluated to determine its efficacy in increasing knowledge about menstrual cycle and management of premenstrual syndrome (PMS) though home remedies with simple kitchen herbal recipes, diet control and breathing exercise along with education of anatomy and physiology or female reproductive system. As calculated approx 6 in 10 women in Europe suffer from PMS where as in India the rate of PMS is at higher level, 159,760,591 against 1,065,070,607 populations which is 9 in 10 [1]. With the highest adolescent population in India, the real challenge for the nation is to provide nutritional health and educational needs for this segment of the population, particularly girls, according to the recent report released by UNICEF [2]. In India one-third adolescent girls are under nourished. Survey reports indicate an approximation of 56.2% of girls to be under nourished and the rate of literacy to be 53.87%, thus indicating considerable ‘unmet needs’ in terms of education, health and nutrition [3].

## Aim

To assess the effectiveness of structured teaching program on knowledge, attitude and practice regarding, premenstrual syndrome and its management among teenagers in high school at Bangalore.

## Objectives

To assess the knowledge regarding premenstrual syndrome among teenagers in terms of pre-test score and to evaluate the effectiveness of structured teaching programme by comparing pre-test and post-test score along with it to find out the association between score of knowledge, attitude and practice and the selected demographic variables.

## Method

Subjects consisted of students (experimental group: 2134, control group: 2134). The experimental group participated in a PMS nutritional education program for 8 weeks (including group and individual involvement). Data was collected before and after the education, and measurement tools were premenstrual symptoms, PMS knowledge, and self health behaviour.

## Results

After the intervention, the experimental group showed a significant increase in PMS knowledge (Z=6.32, p=.000) and self health behavior (t=3.00, p=.004) compared to the control group. After the intervention the experimental group showed a significant increase in PMS knowledge (Z=-4.64, p=.000) and self health behavior (t=-3.04, p=.005) than before the intervention.

## Conclusions

These results suggest that the short term effects of a PMS nutritional education programme for teenagers (Studying in Bangalore district) was proven useful and the program should be applied to PMS nutrition education for PMS clients as well as health professionals.

## Recommendations

Individual health assessment and counselling along with home herbal remedies, diet chart, exercise and religious counselling for prevention of PMS among teenagers.

**Figure 1:**